# Supplementary material for: mTOR Inhibitors as Radiosensitizers in Neuroendocrine Neoplasms
Source: Front Oncol. 2021 Feb 8;10:578380. doi: 10.3389/fonc.2020.578380 (PMC7897674; doi:10.3389/fonc.2020.578380)
Supplement: Supplementary file 1 [file DataSheet_1.pdf]

Supplementary Figures

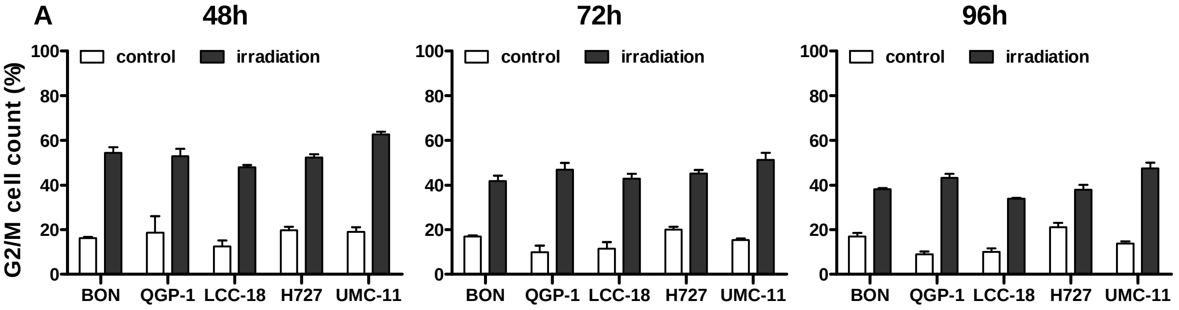

Figure S1 | Irradiation leads to G2/M accumulation of NEN cells after 48, 72 and 96 h. NET cell lines were irradiated with 10 Gy and samples were collected after the indicated time points for cell cycle analysis by flow cytometer. Data are shown as bar diagrams with mean ± S.E.M. (n=2-3).

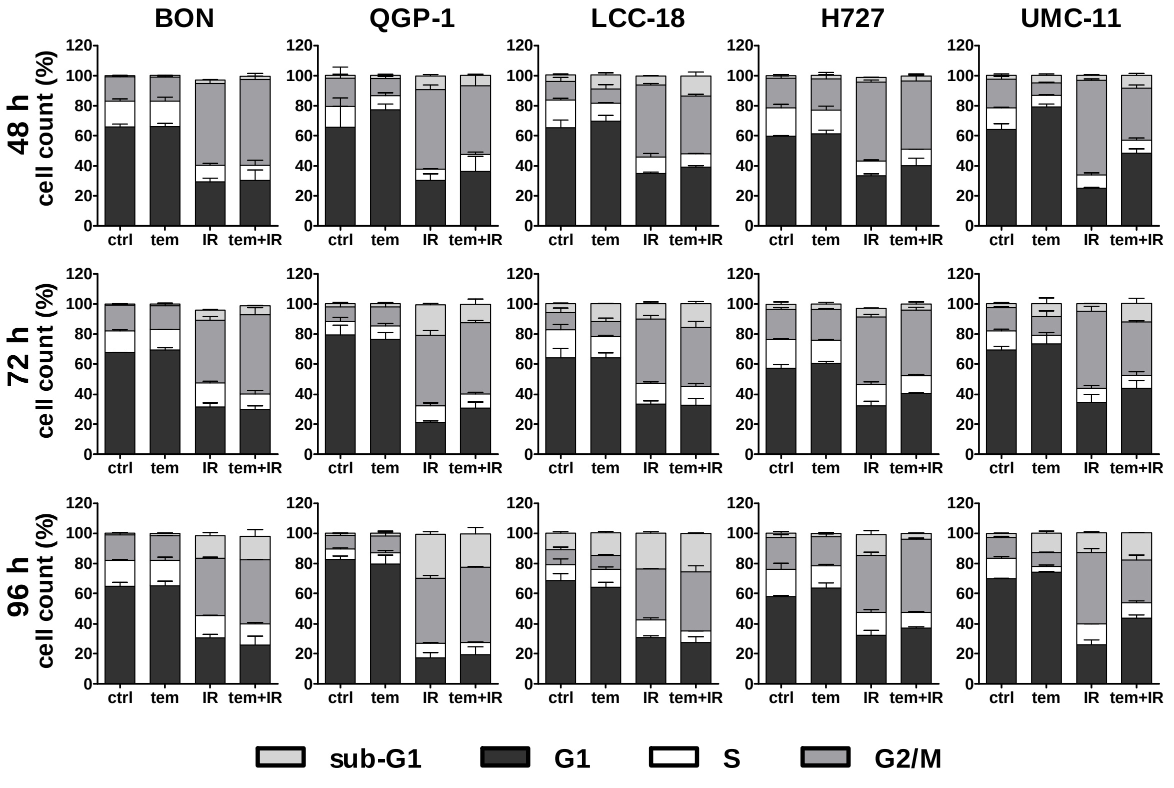

Figure S2 | Cell cycle distribution at 48, 72 and 96 h after combined treatment. NEN cell lines were incubated with 1 µM temsirolimus (tem) or vehicle (ctrl) for 24 h before irradiation. For assessment of cell cycle distribution pretreated NEN cells were collected 48, 72 or 96 h after irradiation with 10 Gy (IR, tem+IR), stained with propidium iodide and analyzed by flow cytometer. Data show mean ± S.E.M. (n=2-3).

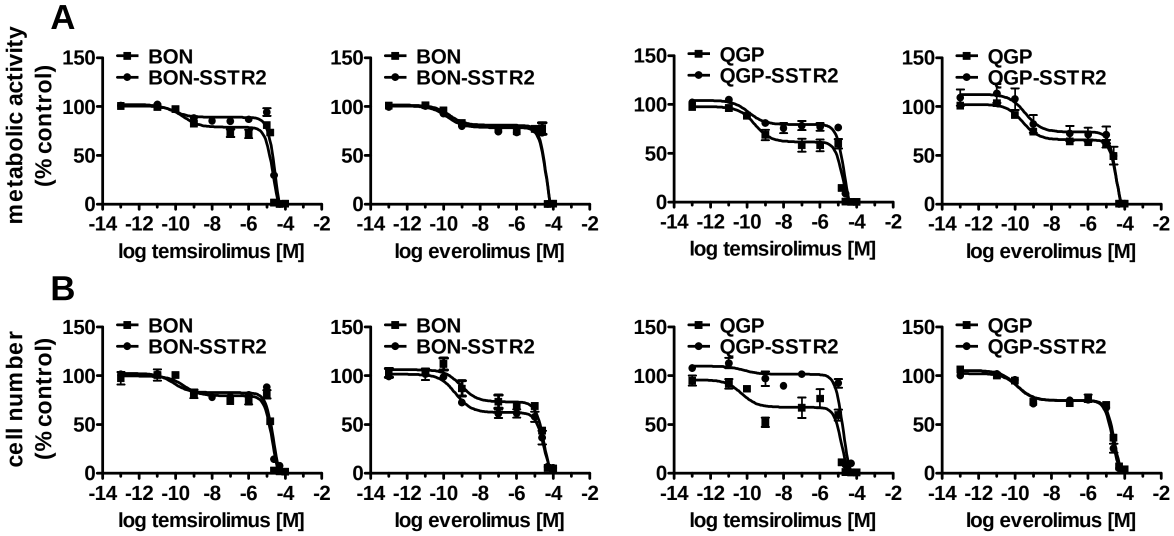

Figure S3 | SSTR2 transfection did not alter sensitivity for mTOR inhibitor treatment. Wild-type or SSTR2-transfected NEN cell lines were treated with increasing concentrations of temsirolimus or everolimus (0.1 pM to 100 µM), incubated for 96 h and analyzed for metabolic activity (A) and cell number (B). Data represent mean ± S.E.M. (n=3).
